# Phytochemicals of nutraceutical importance from different pear cultivars in the early stage of development

**DOI:** 10.1016/j.fochx.2024.102047

**Published:** 2024-11-27

**Authors:** Abdul Basit, Abdul Mueed, Li Min, Niu Mingxu, Gong Xin, Raheem Shahzad, Wen Yue, Tian Jia, Tao Shutian

**Affiliations:** a Sanya Institute, College of Horticulture, Nanjing Agricultural University, Nanjing, China.; b State Key Laboratory of Food Science and Technology, Nanchang University, Nanchang, Jiangxi, China; c Department of Horticulture, The University of Haripur, Haripur, KPK, Pakistan; d College of Horticulture, Xinjiang Agricultural University, Urumqi, China

**Keywords:** Pear, Bioactive compounds, HPLC, Molecular docking

## Abstract

To make full use of young pear fruit thinned from the trees for optimal fruit load during cultivation, this study explored the nutritional diversity in young fruit of seventy-nine different pear varieties, focusing on their bioactive compounds. Our results showed significant variability in total phenolic content (TPC), total flavonoid content (TFC), and antioxidant activity of pear varieties. The TPC values ranged from 0.317 ± 0.051 mg GAE/g to 0.0054 ± 0.021 mg GAE/g FW; the highest TPC value has been found in Lixian new bapan, mulberry pear, and red pear varieties, while the lowest value has found in yaqing, weining fragrant pear and apple pear varieties. Similarly, the TFC values demonstrated substantial differences, with Lijiang sesame pear (0.16 ± 0.01), Lixian new bapan (0.13 ± 0.04), and Xiangyuan (0.13 ± 0.02) pear exhibiting the highest flavonoid content. Antioxidant activity, assessed using the Ferric Reducing Antioxidant Power (FRAP) assay, varied significantly, indicating diverse phytochemical profiles across the varieties. HPLC analysis showed that the high value of bioactive compounds is chlorogenic acid (17.86 ± 4.5), arbutin (2.57 ± 0.3), Epicatechin (1.57 ± 0.27), rutin (0.04 ± 0.03) and ferulic acid (0.30 ± 0.04) found in the mulberry pear variety. Molecular docking studies revealed that chlorogenic acid, Epi-catechin, Rutin, and Ferulic acid showed strong affinity towards proteins such as Nrf2, NF-κB, and iNOS, suggesting potential health benefits. These findings provide valuable insights for breeders, nutritionists, and the food industry, emphasizing the importance of the nutritional quality of pear fruits, and their recycling utilization in the production practice.

## Highlights

• Seventy-nine pear varieties revealed a significant difference in their biochemical constituents
• Various pear varieties contain significant amounts of bioactive compounds.
• Pear varieties revealed significant differences in their antioxidant potential.
• Molecular docking studies revealed strong affinity of compounds towards various proteins.

## Graphical abstract

**Figure:**
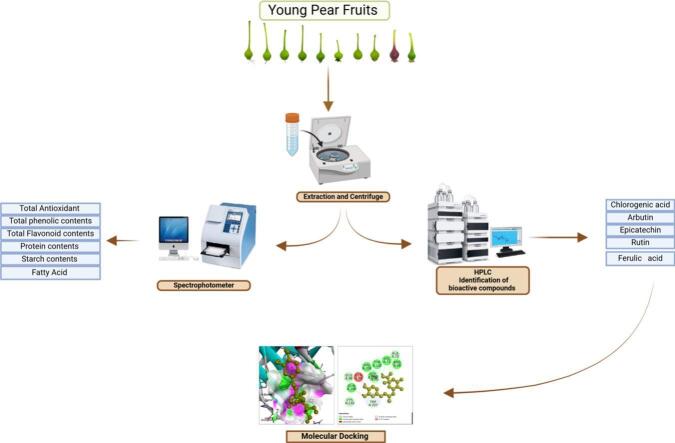

## 1 Introduction

*Pyrus* L. is one of the most widely cultivated fruits in temperate regions of China, followed by apples and grapes in terms of planting area and fruit production (Meng et al., 2021). China is the world's largest producer of pears, accounting for more than 60 % of global pear production. Pears is one of the top two consumed pome fruit among adults, behind apples, according to the extensive European food consumption database, with daily intake ranging from 23 to 108 g (Authority, 2011). Different varieties of pears fruit have consistently included various phytochemicals, such as arbutin, oleanolic acid, ursolic acid, chlorogenic acid, rutin, and epicatechin (Nieman et al., 2015), especially higher in young fruits. These bioactive compounds exhibit diverse biological activities, including potent antioxidant, anti-inflammatory, and antimicrobial effects, making pears a valuable source for nutraceutical applications (Ulaszewska et al., 2018). A study analyzed the organic acid composition of fruits from 40 pear cultivars across four species in China. Fructose was identified as the dominant sugar, with glucose and sucrose in lesser amounts. The ‘Dangshan’ pear had the highest organic acid content at 3607 mg/kg, followed by the ‘Nanguo’ at 2564 mg/kg and the ‘Kuerle’ fragrant pear at 2171 mg/kg. The ‘Kuerle’ pear also had a higher sugar-to-acid ratio. Among the pear varieties, ‘Dangshan’ had the highest total amino acid concentration at 278 mg/100 g. Pear peels had more dietary fiber (21.3 mg/g FW) than the pulp and were more prosperous in quality, containing beneficial substances like flavonoids and polyphenols (Simmonds & Preedy, 2015).

Pear fruits, particularly peels, have a high amount of total phenol with significant antioxidant activity (Michailidis et al., 2021), and are linked to protection against oxidative damage (Qiu et al., 2018). Temperature affects the volatile like ester, aldehydes, alcohols, and ketones chemicals component of the scent of pear fruit (Yao et al., 2018). Some *P. communis* L. varieties of mature fruit had totally or partially red skin or were cleaned, depending on horticultural, genetic, and environmental conditions (Thomson et al., 2018). For the genus *Pyrus,* many domesticated organisms that provide rich germplasm have been selected by long-lasting anthropogenic and environmental pressure (Piluzza et al., 2023b). Historically, stray trees beside highways, in cultivated fields, and on the margins of farms were trained and grafted with local kinds that were early ripening or more prolific.

In most fruits, the ripening process involve a series of coordinated biochemical and physiological changes. Previous studies have investigated alteration in specific food components throughout development. Physiological abscission of immature fruits is a natural process that typically occures at two distinct stages. The initial fruit drop is often attributed to degeneration, incomplete pollination, ovual malformation or nutrient deficiency, while the second drop results from endogenous hormone levels associated with early embryo maturation. These abscised immature fruits are generally small, young and green detaching either from stem-branch or ovary stem junction due to physiological triggers. Notably, the immature fruits contains a significant levels of phytochemicals, such as flavonoids, limonoids, and synephrine, along with higher antioxidant activity compared to mature fruits. Therefore, in this study we analyzed the nutraceutical potential of immature pear fruits.

Considerable research has focused on incorporating fruit waste into extruded snacks, short-dough biscuits, yogurt, kefir, and functional cookies to developed novel functional food. Recovering phenolic compounds from discarded unripe fruits is essential, especially in the search for sustainable and cost-effective bioactive compounds that enhance nutritional value and serve as natural food colorants in various food matrices. Despite the vast quantities of waste generated by the food industry, seasonal production cycles and the variable composition of waste materials presents the major challenges to their industrial application. However, to the best of our knowledge, limited research has focused on the potential applications of immature pear fruits nutritional value, phytochemical composition and biological activity.

The indigenous cultivars of pears in China are pretty variable in terms of several agronomic characteristics, including fruit size, ripening time, soil adaptation, and tolerance to weather conditions, which are typified by high summer temperatures and limited summertime water availability (Piluzza et al., 2023a). Therefore, we have grown more than seventy-nine varieties of pear fruit to discriminate these varieties based on their genetic, nutritional potential, agronomic growth, and yield of fruits. The current study aimed to assess the TPC, TFC, phenolic compounds, antioxidant properties, and nutritional profile of seventy-nine immature pear fruits. Furthermore, we analyzed the molecular docking to understand the molecular importance of pear fruit bioactive compounds.

## 2 Materials and methods

### 2.1 Chemicals and reagents

The chemical reagents and reference standards for HPLC, including Chlorogenic acid, Arbutin, Epicatechin, Rutin, Ferulic acid, Folin and Ciocalteu reagent, Gallic acid, Methanol, 2′-diphenyl-1-picrylhy-drazyl (DPPH), and acetonitrile used in this study were purchased from the MACKLIN (Shanghai Yuanleaf biological technology Co., Ltd., China). All the chemicals and reagents used in this study were of research grade.

### 2.2 Sample collection

**Fig. 1:**
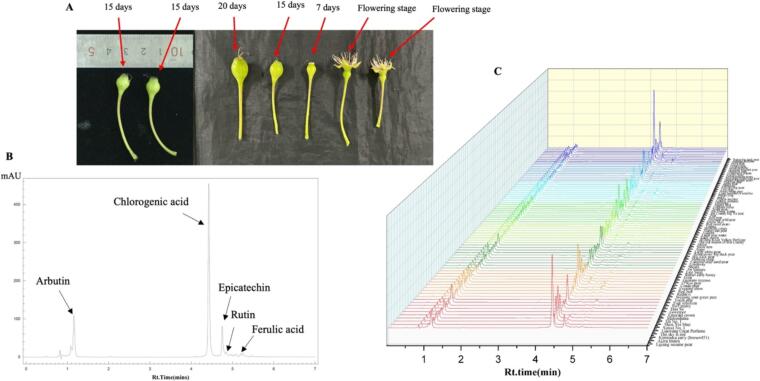
A) Different developing stage of pear fruit. B) HPLC analysis of phytochemicals. C) HPLC analysis of phytochemicals in seventy-nine different pear fruits varieties.

The young pear fruits were obtained from the ‘Hushu’ germplasm resource nursery in Nanjing City, Jiangsu Province, China. A total of seventy-nine accessions of young pear fruits were analyzed in this study. Each variety was uniformly selected from three healthy trees. The Seventy-nine accessions of young pear fruit varieties were collected on the 21st and 22nd of April 2023, 15 days after flowering (Fig. 1A). Each tree was selected uniformly in size, with no disease or pest. Immediately after picking the young pear fruit, they were placed in the ice box and returned to the laboratory for analysis and further storage. The young pear whole fruit pulp was separated, immediately frozen in liquid nitrogen, and stored in falcon tubes at −80 °C until the analysis.

### 2.3 Sample preparation

The extracts were prepared according to the protocol described by Peng et al. (2019) with slight modifications. Briefly, 0.2 g of each young pear variety powder was mixed with 5 mL of 80 % methanol and homogenized with Bead mill Homogenizer at 4.00 m/s speed for 3 min twice and subjected to an ultrasonic cleaner for 30 min. After ultrasonic, the young pear extract was centrifuged (Eppendorf, Germany) at 4500 rpm for 10 mins under 4 °C. The supernatants were collected and stored at −20 °C before further analysis.

### 2.4 Determination of protein

Protein contents in young pear fruits were determined using the “Protein Content Kit” of Suzhu Geruisi Biotechnology Co., Ltd., China. Briefly, 0.1 g of the young pear fruit sample was ground in 1 mL distilled water, homogenized in an ice bath, and the supernatant was transferred to an Eppendorf (EP) tube. The EP tubes were centrifuged at 12000 rpm at room temperature for 10 min, and the resulting supernatant was collected in the new EP tubes. The reaction mixture for protein contents determination was sample 40 μL, working solution 200 μL. The Blank sample used 40 μL distilled water and 200 μL working solution. After mixing, the EP tubes were allowed to cool at room temperature for 10 min. The reaction mixture was transferred to a 96-well microliter plate, and the absorbance was read at 600 nm with a spectrophotometer. Each treatment was replicated thrice, and the experiment was repeated twice.

### 2.5 Determination of free fatty acid

Free fatty acid contents in young pear fruits were determined using the “Free Fatty Acid Content Kit” of Suzhu Geruisi Biotechnology Co., Ltd., China. Briefly, 0.2 g of the young pear fruit sample was crushed in liquid nitrogen with a pestle and mortar, and then 1 mL extraction solution was added, vortexed, and the supernatant was transferred to an Eppendorf Tube. The extract was placed for shaking on a shaker for 3 h. After shaking, the EP tubes were centrifuged at 8000 rpm at room temperature for 10 min, and the supernatant was collected in the new EP tubes. The reaction mixture for free fatty acid contents determination was sample 400 μl, reagent one 200 μl. For the blank sample, 400 μl sample and 200 μl reagent 2. After 5mins mixing, the EP tubes were incubated at room temperature for 5 mins. The reaction mixture (200 μl) was transferred to a 96-well microtitre plate, and the absorbance was read at 715 nm with a spectrophotometer. Each treatment was replicated thrice, and the experiment was repeated twice.

### 2.6 Determination of starch

Starch contents in young pear fruits were determined using the “Starch Content Kit”, of Suzhu Geruisi Biotechnology Co., Ltd., China. Briefly, 0.1 g of the young pear fruits sample was crushed and minced in liquid nitrogen with a pestle and mortar, and then 1 mL reagent was added, vortexed, and the supernatant was transferred to an Eppendorf Tube. The Eppendorf tubes were incubated in a water bath at 50 °C for 30 min, mixing every 3 min. The EP tubes were centrifuged at 10000 rpm at room temperature for 5 min, and the resultant supernatant was collected in the new EP tubes. Further, 0.5 mL distilled water was added to the samples and incubated in a water bath at 95 °C for 15 mins (EP tube covers were tightened with paraffin so as not to escape liquid loss). After 15 min incubation, the samples were allowed to cool at room temperature. After cooling, 0.35 mL concentrated hydrochloric acid was added and incubated at room temperature for 15 mins, followed by adding 0.85 mL distilled water. The EP tubes were centrifuged at 10000 rpm at room temperature for 10 min. The reaction mixture for starch contents determination was as follows: sample 50 μl, working solution 250 μl (prepared by adding 3.75 mL of distilled water in reagent 2, then slowly adding 11.25 mL of concentrated sulfuric acid). For the blank sample, 400 μl sample and 200 μl reagent 2. After 5mins mixing, the EP tubes were incubated at room temperature for 5 mins. After mixing, the EP tubes were put in a water bath at 95 °C for 10 mins (EP tube covers were tightened with paraffin to avoid liquid loss) and allowed to cool at room temperature. The reaction mixture (200 μl) was transferred to a 96-well microtitre plate, and the absorbance was read at 620 nm with a spectrophotometer. Each treatment was replicated thrice, and the experiment was repeated twice.

### 2.7 HPLC analysis

The HPLC apparatus carried out the identification and quantitative determination of phytochemicals in the young pear fruit extract. The Chromatographic system was equipped with a UV-VIS detector. Separation was performed by ACE 5C-18 column (250 mm × 4.6 mm id, 5 μm particle size). During the mobile phase, 90 % water (H_2_O) and 10 % methanol (MeOH) were mixed. The flow rates were 1.0 mL/min, and the injection volume was 20 μL. The column temperature was kept at 30 °C, and detection was carried out at 280 nm. The mixture of 80 % methanol (MeOH) and 20 % deionized water was used to dilute the extract, and the solution was diluted with Bear ruptor elite or bead mill homogenizer machine at 4.00 m/s speed for 3 min after the dilution samples were subjected to an ultrasonic cleaner for 30 min (2 times). After the ultrasonic cleaner, the samples were centrifuged at 4500 rpm for 10 min at room temperature (2 times). After centrifuging, the samples of each variety were separated into 5 mL tubes.

### 2.8 Phenolic and antioxidant assay

#### 2.8.1 Total phenolic content (TPC)

The TPC of young pear samples was determined by the spectrophotometric method described by (Samsonowicz et al., 2019)with some modifications. Briefly, the sample extracts (25 μL), Folin-Ciocalteu reagent solution (25 μL, 1:3 diluted with water), and Milli-Q water (200 μL) were added to a 96-well plate. After incubation (25 °C, 5 mins), 25 μL 10 % (w:w) sodium carbonate was added and followed by incubation in the dark for 60 min. The absorbance was determined at 764 nm in a microplate reader. The quantification of each sample was based on the standard curve generated with 0-200 μg/mL gallic acid in ethanol. The result was expressed as mg of gallic acid equivalents per gram fresh weight of the sample (mg GAE/g of FW).

#### 2.8.2 Total flavonoid content (TFC)

The sample of young pear extract (80 μL), 2 % aluminum chloride (80 μL, *w*/*v*, diluted with ethanol), and sodium acetate solution (120 μL, 50 g/L) were mixed in a 96-well plate and then incubated in the dark at room temperature for 60 mins. The absorbance was measured at 440 nm in a microplate reader. The calculation of TFC of each sample was based on the standard curve of quercetin (0 to 50 μg/mL), and the result was expressed as mg of quercetin equivalent per g (mg QE/g FW) of fresh weight.

#### 2.8.3 Antioxidant activity

Ferric ion reducing capacity (FRAP) in the seventy-nine young pear fruit varieties was estimated according to the described method by Natić et al. (2015). In brief, 20 μL of sample solution of young pear fruit was added into a 96-well microplate, and 200 μL of FRAP working solution was mixed and shaken at 300 r/min for 10 mins at 37 °C. The absorbance was read at 593 nm using an enzyme maker. Trolox as a standard was used, and the results were expressed as μmol TE/g FW.

### 2.9 Molecular docking

The potential interactions of ligands from various young pear cultivars were investigated using the Autodock program version 4.2. The receptor protein was prepared by removing water molecules, adding hydrogen atoms, and assigning partial charges based on the CHARMM force field. Ligand structures were minimized using the MMFF94 force field, with other parameters set to default. Finally, the docking protocol was applied to the processed protein and ligand structures as previously described (Kim et al., 2020).

### 2.10 Statistical analysis

All the experiments were performed in triplicate, and results were presented as mean ± standard deviation (SD) per gram fresh weight, representing commercial young pear fruits and their replicates. We conducted a one-way ANOVA followed by post hoc analyses using Tukey's test to assess statistical differences between the groups. Statistical significance was set at *p* < 0.05. Multivariate statistical analyses, comprising principal component analysis (PCA) were conducted using Origin 2025. Heatmap and dumbbell plot analysis were conducted using an online website (https://www.chiplot.online).

## 3 Results and discussion

### 3.1 Nutritional composition of different young pear fruits

The proximate analysis included starch, protein, and free fatty acid content measurements, revealed significant variability among the varieties (*p* ≤ 0.05). The contents of free fatty acids showed considerable differences, with values ranging from 3.35 ± 0.03 μmol/g FW to 0.005 ± 0.01 μmol/g FW respectively (Table 1). The highest concentrations were in the varieties sutsui no.1, kinizuka early (brown), early brown, and Jinchuan wild pear. In contrast, the lowest concentrations were detected in foal selection, longevity, and nova varieties. Our result is consistent with the findings of Silva et al. (2021a), who reported that the fat content in *Opuntia ficus indica* was from 0.04 to 0.97 g/100 g, while crude fat content was 0.4 g/100 g. Similarly, Özcan and Al Juhaimi (2011) reported that 5 % crud fat has been found in *Opuntia ficus indica* pear fruit extract. Starch contents varied widely among the young pear fruit varieties, ranging from 4.353 ± 0.163 mg/g FW to 0.006 ± 0.004 mg/g FW, respectively (Table 1). It has been reported that 4 to 7.12 g/100 g of starch has been found in *Opuntia ficus indica* pear (Silva et al., 2021b). Varieties such as August red, Sutsui no.1, Morning crispy, Autumn perfume, and Lijiang sesame pear exhibited the highest starch concentrations, while the red pear, puff pastry, xiangyuan pear, and overwriting pears had the lowest starch contents. The varying starch during fruit development aligns with observations made in other fruits by (Oikawa et al., 2015), suggesting that starch biosynthesis and degradation are critical processes influenced by both developmental stages and genetic factors. Protein content in the studied pear varieties also showed significant variation, ranging from (0.636 ± 0.05 mg/g FW to 0.0013 ± 0.004 mg/g FW, respectively (Table 1). However, higher protein contents were found in Shaydong black sour pear, Atogo, Showa, Eli. no.1, and Weining sour green pear, while lower levels were observed in Liaoyang great perfume, gold pear, Great Perfume, Moon, and Yaqing. These findings align with previously reported data by Li et al. (2018) which documented similar variability in protein content among different pear cultivars, emphasizing the role of genetic diversity in determining protein accumulation. The significant differences in the proximate composition of young pear varieties highlight the importance of genetic factors and developmental stages in determining their nutritional quality. These variations in starch, protein, and free fatty acid content among the pear varieties underscore the complexity of their nutritional profiles and potential health benefits. Understanding these differences is crucial for breeders and food scientists aiming to enhance the nutritional quality of pear fruits. Moreover, this information can guide consumers and industries in selecting specific pear varieties for dietary purposes or processing needs. Further research should explore the underlying biochemical pathways and environmental influences contributing to these variations, potentially leading to improved cultivation practices and enhanced fruit quality.

**Table 1:** The proximate composition, TPC, TFC, and antioxidant properties of different pear fruit varieties.

**Varieties**	**Total Phenolic contents (mg GAE/g FW)**	**Total Flavonoid Contents (mg/g FW)**	**Total Antioxidant (FRAP) (**μmol **Trolox/g FW)**	**Starch contents (mg/g FW)**	**Protein contents (mg/g FW)**	**Fatty Acid (μmol/g FW)**
**Lijiang sesame pear**	0.24 ± 0.16^abcdefgh^	0.16 ± 0.01^ab^	1.31 ± 0.30^a^	2.84 ± 0.29^de^	0.23 ± 0.03^hi^	1.82 ± 0.029^e^
**Akira Imura**	0.21 ± 0.07^abcdefgh^	0.06 ± 0.02^cdefghijkl^	0.64 ± 0.18^cdefghijk^	1.89 ± 0.01^jklm^	0.08 ± 0.003^stuvw^	0.03 ± 0.021^g^
**Kimizuka early (brown451** **)**	0.22 ± 0.01^abcdefgh^	0.08 ± 0.008^bcdefghijkl^	0.55 ± 0.23^cdefghijkl^	1.44 ± 0.02qrstu	0.42 ± 0.02^d^	2.20 ± 0.08^d^
**The sky is red**	0.19 ± 0.004^abcdefgh^	0.1 ± 0.002^bcdefghijkl^	0.44 ± 0.05^efghijkl^	1.94 ± 0.009^ijkl^	0.13 ± 0.02^nopqr^	0.92 ± 0.03^ijklmn^
**Liaoyang Great Perfume**	0.19 ± 0.02^abcdefgh^	0.1 ± 0.01^bcdefghijkl^	0.62 ± 0.10^cdefghijkl^	1.21 ± 0.01^uvwxyz^	0.001 ± 0.004^a^	0.06 ± 0.009^fg^
**Sutsui No. 1**	0.13 ± 0.02^bcdefgh^	0.01 ± 0.002^l^	0.51 ± 0.13^cdefghijkl^	3.69 ± 0.4^1b^	0.14 ± 0.01^mnop^	3.04 ± 0.2^1b^
**Shou Xin Shui**	0.11 ± 0.01^cdefgh^	0.03 ± 0.002^fghijkl^	0.31 ± 0.02^ghijkl^	1.62 ± 0.03^nopqr^	0.43 ± 0.02^d^	0.26 ± 0.02^bcd^
**Eli No. 1**	0.09 ± 0.006^fgh^	0.04 ± 0.03^efghijkl^	0.20 ± 0.04^jkl^	1.09 ± 0.03^xyzab^	0.34 ± 0.01^e^	1.39 ± 0.10^f^
**Matsushima**	0.24 ± 0.07^abcdefgh^	0.11 ± 0.03^bcdefghijkl^	0.88 ± 0.23^abcdef^	1.29 ± 0.01^tuvwx^	0.08 ± 0.005^stuvwx^	0.89 ± 0.08^jklmn^
**Emerald crown**	0.08 ± 0.02^fgh^	0.01 ± 0.005^kl^	0.38 ± 0.15^fghijkl^	2.61 ± 0.22^ef^	0.15 ± 0.01^klmn^	1.37 ± 0.08^f^
**Governor**	0.2 ± 0.15^abcdefgh^	0.04 ± 0.03^efghijkl^	0.61 ± 0.24^cdefghijkl^	2.03 ± 0.01^ij^	0.23 ± 0.02^hi^	0.74 ± 0.07^opqr^
**Hua Su**	0.21 ± 0.01^abcdefgh^	0.06 ± 0.007^cdefghijkl^	0.93 ± 0.11^abcde^	1.82 ± 0.03^jklmno^	0.10 ± 0.01^rstuv^	0.95 ± 0.04^hij^
**Puff pastry**	0.18 ± 0.01^abcdefgh^	0.07 ± 0.006^bcdefghijkl^	0.37 ± 0.02^fghijkl^	0.05 ± 0.03^im^	0.11 ± 0.01^opqrs^	0.51 ± 0.03^vwx^
**Foal selection**	0.26 ± 0.03^abcdefg^	0.09 ± 0.01^bcdefghijkl^	0.76 ± 0.07^bcdefgh^	2.02 ± 0.04^ij^	0.24 ± 0.03^hi^	0.005 ± 0.01^g^
**Torch pear**	0.24 ± 0.12^abcdefgh^	0.13 ± 0.06^bcdef^	0.64 ± 0.17^cdefghijkl^	1.84 ± 0.01^jklmn^	0.13 ± 0.01^nopqr^	1.27 ± 0.2^f^
**Weining sour green pear**	0.2 ± 0.08^abcdefgh^	0.09 ± 0.03^bcdefghijkl^	0.60 ± 0.15^cdefghijkl^	1.39 ± 0.008^rstuv^	0.34 ± 0.006^e^	1.10 ± 0.09^g^
**Redskin**	0.23 ± 0.02^abcdefgh^	0.1 ± 0.02^bcdefghijkl^	0.70 ± 0.04^bcdefghij^	1.98 ± 0.006^ijkl^	0.06 ± 0.01^wxy^	0.53 ± 0.03^uvw^
**Red Sun**	0.08 ± 0.02^fgh^	0.03 ± 0.01^fghijkl^	0.24 ± 0.02^ijkl^	1.98 ± 0.006^hi^	0.25 ± 0.01^h^	0.86 ± 0.03^jklmno^
**Evening show**	0.19 ± 0.006^abcdefgh^	0.05 ± 0.006^defghijkl^	0.71 ± 0.06^bcdefghij^	2.13 ± 0.006^hi^	0.16 ± 0.03^klmn^	0.9 ± 0.08^jklmn^
**Conde pear**	0.09 ± 0.03^fgh^	0.034 ± 0.01^fghijkl^	0.22 ± 0.02^jkl^	2.13 ± 0.008^hi^	0.15 ± 0.01^klmn^	1.14 ± 0.18^g^
**O'Woo pear**	0.09 ± 0.02^fgh^	0.02 ± 0.008^ijkl^	0.36 ± 0.19^fghijkl^	0.61 ± 0.02^fghi^	0.15 ± 0.02^klmn^	0.39 ± 0.06^xyza^
**Gyokuro incense**	0.15 ± 0.02^bcdefgh^	0.07 ± 0.006^bcdefghijkl^	0.44 ± 0.07^defghijkl^	1.69 ± 0.02^mnop^	0.10 ± 0.01^qrstuv^	0.69 ± 0.02^qrst^
**Gion**	0.22 ± 0.05^abcdefgh^	0.07 ± 0.02^bcdefghijkl^	0.96 ± 0.10^abcd^	0.85 ± 0.04^bcde^	0.32 ± 0.01^ef^	1.75 ± 0.1^f^
**Rather early honey**	0.07 ± 0.03^gh^	0.01 ± 0.005^jkl^	0.31 ± 0.007^ghijkl^	1.62 ± 0.04^nopqr^	0.14 ± 0.02^lmno^	0.34 ± 0.03^abc^
**Late Sanji**	0.23 ± 0.02^abcdefgh^	0.056 ± 0.008^defghijkl^	0.65 ± 0.13^bcdefghijk^	2.002 ± 0.02^ijk^	0.14 ± 0.02^mnopq^	0.36 ± 0.04^zabc^
**Jin Shuiqiu**	0.12 ± 0.006^bcdefgh^	0.04 ± 0.01^efghijkl^	0.41 ± 0.05^fghijkl^	0.81 ± 0.01^cdefg^	0.13 ± 0.03^nopqr^	0.29 ± 0.01^abc^
**Showa**	0.19 ± 0.11^abcdefgh^	0.07 ± 0.05^bcdefghijkl^	0.61 ± 0.38^cdefghijkl^	1.39 ± 0.01^qrstuv^	0.44 ± 0.03^d^	0.52 ± 0.02^uvw^
**Longevity**	0.16 ± 0.10^bcdefgh^	0.07 ± 0.05^bcdefghijkl^	0.63 ± 0.37^cdefghijkl^	0.64 ± 0.02^efghi^	0.25 ± 0.03^h^	0.03 ± 0.02^g^
**Cangwu large sand pear**	0.19 ± 0.02^abcdefgh^	0.12 ± 0.03^bcdefgh^	0.52 ± 0.09^cdefghijkl^	2.31 ± 0.02^gh^	0.18 ± 0.009^jkl^	0.19 ± 0.03^de^
**Morning crisp**	0.21 ± 0.07^abcdefgh^	0.09 ± 0.05^bcdefghijkl^	0.54 ± 0.19^cdefghijkl^	1.69 ± 0.01^mnop^	0.07 ± 0.001^tuvwxy^	0.51 ± 0.09^uvw^
**Big duck pear**	0.16 ± 0.05^bcdefgh^	0.08 ± 0.03^bcdefghijkl^	0.63 ± 0.09^cdefghijkl^	2.29 ± 0.08^gh^	0.07 ± 0.004^uvwxy^	1.79 ± 0.11^e^
**Rendezvous big duck pear**	0.11 ± 0.005^defgh^	0.05 ± 0.005^defghijkl^	0.50 ± 0.18^cdefghijkl^	2.75 ± 0.05^de^	0.08 ± 0.001^stuvw^	0.83 ± 0.03^klmnop^
**Large white pear**	0.11 ± 0.06^defgh^	0.04 ± 0.03^defghijkl^	0.32 ± 0.15^ghijkl^	0.75 ± 0.05^defgh^	0.11 ± 0.01^opqrst^	0.82 ± 0.08^lmnop^
**Atago**	0.15 ± 0.04^bcdefgh^	0.05 ± 0.02^defghijkl^	0.57 ± 0.13^cdefghijkl^	0.61 ± 0.02^fghi^	0.56 ± 0.01^b^	0.82 ± 0.04^lmnop^
**Snow new**	0.14 ± 0.04^bcdefgh^	0.04 ± 0.02^efghijkl^	0.50 ± 0.13^cdefghijkl^	1.83 ± 0.06^jklmn^	0.26 ± 0.03^gh^	0.81 ± 0.02^mnopq^
**Moon**	0.1 ± 0.03^defgh^	0.02 ± 0.01^hijkl^	0.32 ± 0.13^ghijkl^	1.63 ± 0.01^nopq^	0.02 ± 0.002^za^	0.54 ± 0.06^uvw^
**The old widow of Bin County**	0.19 ± 0.01^abcdefgh^	0.09 ± 0.008^bcdefghijkl^	0.62 ± 0.06^cdefghijkl^	2.14 ± 0.03^hi^	0.29 ± 0.003^fg^	0.33 ± 0.04^abc^
**Willow River Yellow Perfume**	0.22 ± 0.06^abcdefgh^	0.1 ± 0.02^bcdefghijkl^	0.79 ± 0.17^abcdefg^	1.76 ± 0.01^klmno^	0.13 ± 0.02^nopqr^	0.49 ± 0.03^vwxy^
**Early crown**	0.16 ± 0.03^bcdefgh^	0.04 ± 0.005^defghijkl^	0.61 ± 0.08^cdefghijkl^	2.48 ± 0.12^fg^	0.04 ± 0.005^yz^	2.29 ± 0.03^d^
**Large pear water**	0.18 ± 0.01^abcdefgh^	0.09 ± 0.02^bcdefghijkl^	0.39 ± 0.06^fghijkl^	0.91 ± 0.01^abcd^	0.05 ± 0.01^wxyz^	1.06 ± 0.11^gh^
**Joaquin**	0.14 ± 0.03^bcdefgh^	0.03 ± 0.005^fghijkl^	0.41 ± 0.03^fghijkl^	0.48 ± 0.16^ijk^	0.08 ± 0.01^stuvwx^	0.07 ± 0.05^efg^
**Xiangyuan pear**	0.24 ± 0.008^abcdefgh^	0.13 ± 0.01^bcde^	0.63 ± 0.12^cdefghijkl^	0.26 ± 0.02^ki^	0.13 ± 0.008^nopqr^	0.54 ± 0.05^uvw^
**Morning crispy**	0.14 ± 0.01^bcdefgh^	0.05 ± 0.02^defghijkl^	0.28 ± 0.06^ghijkl^	3.47 ± 0.33^bc^	0.34 ± 0.01^e^	0.59 ± 0.04^tuvw^
**Yaqing**	0.05 ± 0.06^h^	0.01 ± 0.012^l^	0.17 ± 0.07^kl^	0.72 ± 0.008^defgh^	0.002 ± 0.01^a^	0.95 ± 0.03^hijk^
**Red sweet pears**	0.1 ± 0.03^efgh^	0.03 ± 0.006^fghijkl^	0.36 ± 0.09^fghijkl^	0.55 ± 0.03^hij^	0.14 ± 0.01^lmno^	0.52 ± 0.01^uvw^
**Green Skin**	0.27 ± 0.01^abcdefg^	0.12 ± 0.04^bcdefg^	0.64 ± 0.27^cdefghijkl^	0.61 ± 0.02^ghi^	0.36 ± 0.02^e^	0.05 ± 0.07^fg^
**Jinchuan wild pear**	0.1 ± 0.06^efgh^	0.03 ± 0.03^fghijkl^	0.30 ± 0.07^ghijkl^	0.91 ± 0.02^abcd^	0.05 ± 0.01^wxyz^	3.35 ± 0.03^a^
**Red pear**	0.3 ± 0.02^abcd^	0.1 ± 0.02^bcdefghijkl^	0.97 ± 0.26^abc^	0.006 ± 0.004^m^	0.05 ± 0.007^wxyz^	0.06 ± 0.1^fg^
**Pear**	0.18 ± 0.08^abcdefgh^	0.09 ± 0.03^bcdefghijkl^	0.47 ± 0.04^cdefghijkl^	1.78 ± 0.01^klmno^	0.10 ± 0.007^rstuv^	0.84 ± 0.02^jklmnop^
**Jin County big Ya pear**	0.15 ± 0.006^bcdefgh^	0.02 ± 0.002^ghijkl^	0.15 ± 0.006^kl^	0.65 ± 0.03^efghi^	0.13 ± 0.03^nopqr^	0.02 ± 0.04^g^
**De Sheng Xiang**	0.13 ± 0.008^bcdefgh^	0.03 ± 0.03^efghijkl^	0.15 ± 0.006^kl^	1.46 ± 0.009^pqrs^	0.11 ± 0.01^opqrs^	0.33 ± 0.02^abc^
**Sister pear**	0.3 ± 0.09^abcde^	0.11 ± 0.04^bcdefghijkl^	0.60 ± 0.06^cdefghijkl^	0.92 ± 0.03^abcd^	0.35 ± 0.03^e^	0.57 ± 0.01^tuvw^
**Fragrant hemp**	0.17 ± 0.05^bcdefgh^	0.06 ± 0.01^bcdefghijkl^	0.40 ± 0.04^fghijkl^	1.61 ± 0.12^nopqr^	0.14 ± 0.03^mno^	0.37 ± 0.01^yzab^
**August Red**	0.27 ± 0.01^abcdef^	0.11 ± 0.03^bcdefghijkl^	0.60 ± 0.10^cdefghijkl^	4.35 ± 0.16^a^	0.17 ± 0.02^jklm^	2.63 ± 0.08^c^
**Qingsong**	0.11 ± 0.03^cdefgh^	0.04 ± 0.009^efghijkl^	0.26 ± 0.03^hijkl^	3.24 ± 0.76^c^	0.36 ± 0.04^e^	0.03 ± 0.01^g^
**Golden Autumn**	0.15 ± 0.02^bcdefgh^	0.03 ± 0.01^fghijkl^	0.48 ± 0.08^cdefghijkl^	0.53 ± 0.05^hij^	0.002 ± 0.001^a^	0.95 ± 0.04^hijk^
**Yellow incense**	0.09 ± 0.02^fgh^	0.01 ± 0.001^jkl^	0.44 ± 0.19^efghijkl^	1.61 ± 0.08^nopqr^	0.04 ± 0.02^xyz^	0.28 ± 0.03^abcd^
**nova**	0.12 ± 0.003^bcdefgh^	0.04 ± 0.02^efghijkl^	0.35 ± 0.04^ghijkl^	0.01 ± 0.002^m^	0.21 ± 0.009^ij^	0.02 ± 0.35^g^
**Hang Qing**	0.12 ± 0.07^bcdefgh^	0.04 ± 0.04^efghijkl^	0.30 ± 0.19^ghijkl^	1.35 ± 0.02^stuvw^	0.18 ± 0.007^jk^	1.02 ± 0.05^ghi^
**Long handful of ennifies**	0.119 ± 0.02^cdefgh^	0.03 ± 0.01^efghijkl^	0.27 ± 0.04^hijkl^	3.44 ± 0.72^c^	0.06 ± 0.01^wxy^	0.93 ± 0.04^hijklm^
**Small white pear**	0.165 ± 0.03^bcdefgh^	0.05 ± 0.004^defghijkl^	0.30 ± 0.07^ghijkl^	1.04 ± 0.08^yzabc^	0.06 ± 0.03^wxy^	0.53 ± 0.01^uvw^
**Two slots**	0.17 ± 0.12^bcdefgh^	0.07 ± 0.06^defghijkl^	0.48 ± 0.3^cdefghijkl^	1.42 ± 0.02^qrstu^	0.16 ± 0.004^klmn^	0.48 ± 0.03^wxyz^
**Linxia crisp pear**	0.18 ± 0.10^abcdefgh^	0.09 ± 0.04^bcdefghijkl^	0.31 ± 0.13^ghijkl^	1.23 ± 0.02^tuvwxyz^	0.13 ± 0.03^nopqr^	0.6 ± 0.02^stup^
**Zhongcui**	0.18 ± 0.10^abcdefgh^	0.01 ± 0.001^bcdefghijkl^	0.31 ± 0.11^ghijkl^	1.003 ± 0.02^zabc^	0.14 ± 0.04^mnop^	0.74 ± 0.02^opqr^
**Apple pear**	0.08 ± 0.04^fgh^	0.05 ± 0.03^kl^	0.21 ± 0.09^jkl^	1.29 ± 0.03^tuvwx^	0.11 ± 0.005^opqrs^	0.8 ± 0.02^nopq^
**Old man pear**	0.17 ± 0.03^bcdefgh^	0.04 ± 0.008^defghijkl^	0.47 ± 0.04^cdefghijkl^	1.25 ± 0.07^tuvwxy^	0.34 ± 0.008^e^	0.73 ± 0.03^opqr^
**Frozen fragrant pears**	0.23 ± 0.05^abcdefgh^	0.11 ± 0.02^bcdefghi^	0.47 ± 0.20^cdefghijkl^	1.82 ± 0.03^jklmn^	0.12 ± 0.04^nopqr^	0.63 ± 0.04^rstu^
**Shaydong black sour pear**	0.08 ± 0.01^fgh^	0.03 ± 0.004^fghijkl^	0.27 ± 0.07^hijkl^	0.63 ± 0.03^efghi^	0.63 ± 0.05^a^	0.94 ± 0.03^hijkl^
**Overwintering pears**	0.133 ± 0.01^bcdefgh^	0.02 ± 0.002^hijkl^	0.39 ± 0.11^fghijkl^	0.32 ± 0.02^jk^	0.20 ± 0.008^ij^	1.1 ± 0.03^g^
**Green Cloud**	0.62 ± 0.11^bcdefgh^	0.05 ± 0.04^defghijkl^	0.397 ± 0.29^fghijkl^	1.84 ± 0.11^jklmn^	0.16 ± 0.02^klmn^	0.25 ± 0.02^bcd^
**Lixian new Bapan**	0.317 ± 0.048^ab^	0.13 ± 0.02^bcdef^	0.16 ± 0.06^kl^	1.63 ± 0.01^nopqr^	0.23 ± 0.03^hi^	0.02 ± 0.14^g^
**Mulberry pear**	0.313 ± 0.01^abc^	0.1 ± 0.03^bc^	0.87 ± 0.08^abcdef^	1.17 ± 0.11^vwxyz^	0.51 ± 0.04^c^	0.73 ± 0.05^pqrs^
**Weining fragrant pear**	0.05 ± 0.02^h^	0.01 ± 0.01^jkl^	0.12 ± 0.03^l^	0.73 ± 0.03^defgh^	0.23 ± 0.023^hi^	0.63 ± 0.04^rstu^
**Great perfume**	0.248 ± 0.01^abcdefgh^	0.12 ± 0.01^bcdefg^	0.63 ± 0.11^cdefghijkl^	1.58 ± 0.05^opqrs^	0.002 ± 0.002^a^	0.24 ± 0.09^cd^
**Golden pear**	0.089 ± 0.006^fgh^	0.02 ± 0.03^hijkl^	0.29 ± 0.22^ghijkl^	1.74 ± 0.01^lmno^	0.07 ± 0.01^vwxy^	0.67 ± 0.02^rst^
**Green pear**	0.13 ± 0.06^bcdefgh^	0.07 ± 0.04^bcdefghijkl^	0.60 ± 0.12^cdefghijkl^	0.8 ± 0.02^cdefg^	0.05 ± 0.02^wxy^	0.2 ± 0.05^d^
**Gold pear**	0.135 ± 0.01^bcdefgh^	0.03 ± 0.01^fghijkl^	0.603 ± 0.2^cdefghijkl^	1.13 ± 0.01^wxyza^	0.004 ± 0.01^a^	0.17 ± 0.02^def^
**Autumn perfume**	0.204 ± 0.05^abcdefgh^	0.1 ± 0.03^bcd^	0.75 ± 0.17^bcdefghi^	2.95 ± 0.18^d^	0.10 ± 0.01^pqrstu^	0.8 ± 0.04^nopq^
**Botou big duck pear**	0.14 ± 0.05^bcdefgh^	0.06 ± 0.02^bcdefghijkl^	0.37 ± 0.12^fghijkl^	0.85 ± 0.01^bcdef^	0.11 ± 0.012^opqrs^	0.85 ± 0.02^jklmnop^

### 3.2 Phenolic compounds in young pear fruits

The HPLC analysis was performed to assess the presence of various constituents in the young pear fruit extract, and the result of the HPLC analysis is shown in Table 1 and Fig. 1B and C. The 5 standards, such as Chlorogenic acid, Arbutin, epicatechin, Rutin, and Ferulic acid, were run with pear extract to detect the desired compound. In the present study, the quantification of phenolic compounds was carried out by comparing retention times with HPLC-grade reference standards. Five phenolic compounds, including phenolic acids and flavonoids (chlorogenic acid, arbutin, epicatechin, rutin, and ferulic acid), were quantified through HPLC analysis. The Lijiang sesame pear, Sky red, Xiangyuan pear, Mulberry pear, and Autumn perfume varieties exhibited the highest phenolic acid contents. In contrast, the lowest concentrations of chlorogenic acid were observed in the Emerald crown, Conde pear, Jin County big Ya pear, Yellow incense, and Nova varieties (Table 1). Previously, Wang et al. (2021) reported chlorogenic acid as the dominant phenolic compound in several Lithuania- and Sinop-grown pear varieties, including Conference, Concordia, Grabova, and Patten. Additionally, protocatechuic acid was quantified by Truong et al. (2017) through HPLC analysis in Asian-grown pear varieties (*Pyrus* spp.). Tanrıöven and Ekşi (2005) also quantified chlorogenic and caffeic acid in pear juice using the HPLC method in several pear varieties, including Williams, Santa Maria, and Starkrimson. Similarly, arbutin was also found in higher concentrations than epicatechin, rutin, and ferulic acid. The high concentration of arbutin was found in Matsushima, Governor, Foal selection, and red pear varieties. In contrast, a low concentration of arbutin was found in Apple pear, Shaydong black sour pear, Emerald crown, and Red Sun varieties. Other compounds such as epicatechin, rutin, and ferulic acid were found in deficient concentrations in all pear varieties. In a previous study, Brahem et al. (2017) quantified epicatechin in the flesh and peel of 16 European-grown pear varieties, such as Rochas, William Rouge, and William Vert. They found that its concentration was higher in the peel than in the flesh. However, Arts et al. (2000) reported no epicatechin gallate was detected in two Netherlands-grown pear varieties, Conference and Doyenne du Comice, possibly due to differences in pear varieties, growing regions, and extraction solvents.

### 3.3 TPC in different young pear fruits

The TPC values, calculated using the gallic acid standard curve, showed substantial variability among the varieties, ranging from 0.317 ± 0.051 mg GAE/g to 0.0054 ± 0.021 mg GAE/g FW. This range indicates a notable diversity in phenolic content among the pear varieties at the young fruit stage. Among the varieties studied, Lixian new bapan, mulberry pear, red pear, and sister pear exhibited the highest TPC values. These varieties might possess potent antioxidant properties, as phenolic compounds are known for their ability to scavenge free radicals and provide health benefits. A previous study demonstrated that immature peach fruit possesses elevated TPC and enhanced antioxidant activity (Dzah, 2014). On the other hand, the lowest TPC values were found in weining fragrant pear, apple pear, golden pear, yellow pear, and emerald crown. This wide range in TPC underscores the genetic and environmental factors influencing phenolic content in pears. The observed decline in TPC after 15 DAF aligns with a previously reported study, a decrease in phenolic content during fruit development and ripening in various fruits, including pears, suggesting that phenolic biosynthesis might slow down as the fruit matures (Wu et al., 2013). Similarly, (Zhang et al., 2022) found that environmental factors such as light exposure and temperature significantly affect phenolic content in fruits.

These findings have practical implications for agricultural practices and the food industry. Understanding the phenolic content at different developmental stages can guide harvest times to maximize health benefits. Additionally, these results can aid breeders in selecting varieties with higher phenolic content for cultivation, contributing to the production of nutritionally superior fruits. In conclusion, the significant differences in TPC among the seventy-nine pear varieties and the observed decreasing trend during the young stage highlight the complexity of phenolic compound accumulation in pears. Further research is warranted to explore the genetic and environmental determinants of TPC in pears, which could lead to improved cultivation practices and enhanced fruit quality.

### 3.4 Total flavonoid content in different young pear fruits

TFC values highlight the variability in flavonoid accumulation (Table. 2), which can be attributed to genetic diversity and environmental influences. The highest TFC was found in Lijiang sesame pear, Lixian new bapan, and Xiangyuan pear, with values ranging from 0.16 ± 0.01 to 0.13 ± 0.02 mg/g FW. These varieties may offer enhanced health benefits due to their higher flavonoid content, as flavonoids are known for their antioxidant properties. Conversely, the lowest TFC values were observed in varieties such as Sutsui no.1, Emerald crown, Rather early honey, Yaqing, Yellow incense, Zhongcui, and Weining fragrant pear, indicating a significant range in flavonoid content among the different varieties. Similar to this, (Patricia et al., 2020) and (Wang et al., 2021) confirmed that the TFC in pears can vary significantly depending on the extraction solvents used, including n-hexane, ethyl acetate, ethanol, and methanol. These studies suggest that the pear varieties' genetic makeup and the methods used to extract flavonoids are critical factors influencing TFC measurements. The significant differences in TFC among the young pear varieties underscore the complexity of phytochemical compositions within pear species. These findings are valuable for breeders aiming to develop varieties with higher flavonoid content and for nutritionists focusing on the health benefits of pear consumption. Furthermore, understanding the factors influencing flavonoid content can lead to better cultivation practices and more targeted breeding programs. In conclusion, the study highlights the substantial variability in total flavonoid content among seventy-nine Chinese local young pear varieties. This variability is influenced by both genetic factors and extraction methods, as corroborated by previous research. The insights gained from this study provide a foundation for further research into the genetic and environmental factors affecting flavonoid content in pears, ultimately contributing to improved dietary and health benefits.

**Table 2:** HPLC analysis of phenolic compounds in different pear fruit varieties.

**S.no**	**Varieties**	**Chlorogenic acid**	**Arbutin**	**Epicatechin**	**Rutin**	**Ferulic acid**
1	Lijiang sesame pear	12.67 ± 7.30^ab^	3.36 ± 1.90^abcdefghi^	2.23 ± 1.13^a^	0.14 ± 0.15^abcd^	0.24 ± 0.20^defghijk^
2	Akira Imura	3.71 ± 1.67^dfghi^	3.77 ± 1.08^abcdef^	0.61 ± 0.30^cdefghijklm^	0.05 ± 0.009^bcd^	0.14 ± 0.02^ghijk^
3	Kimizuka early (brown451)	6.40 ± 0.04^bcdefghi^	2.68 ± 0.08^abcdefghi^	0.80 ± 0.05^cdefghijk^	0.11 ± 0.008^abcd^	0.17 ± 0.04^ghijk^
4	The sky is red	9.08 ± 0.54^bcde^	2.06 ± 0.08^cdefghi^	0.84 ± 0.02^cdefghij^	0.05 ± 0.01^bcd^	0.15 ± 0.001^ghijk^
5	Liaoyang Great Perfume	5.97 ± 0.63^bcdefghi^	2.20 ± 0.13^bcdefghi^	0.31 ± 0.04^ghijklm^	0.04 ± 0.01^bcd^	0.17 ± 0.02^ghijk^
6	Sutsui No. 1	0.47 ± 0.13^i^	3.12 ± 0.86^abcdefghi^	0.04 ± 0.01^m^	0.06 ± 0.01^bcd^	0.10 ± 0.02^ghijk^
7	Shou Xin Shui	2.41 ± 0.22^efghi^	1.66 ± 0.16^defghi^	0.11 ± 0.007^klm^	0.03 ± 0.01^bcd^	0.20 ± 0.14^fghijk^
8	Eli No. 1	1.53 ± 0.61^ghi^	1.19 ± 0.33^efghi^	0.08 ± 0.03^lm^	0.02 ± 0.01^cd^	0.14 ± 0.05^ghijk^
9	Matsushima	6.96 ± 1.03^bcdefghi^	5.23 ± 0.34^a^	0.25 ± 0.05^hijklm^	0.11 ± 0.02^abcd^	0.60 ± 0.46^bcdef^
10	Emerald crown	0.57 ± 0.29^i^	0.78 ± 0.53^ghi^	0.04 ± 0.02^m^	0.03 ± 0.008^cd^	0.03 ± 0.02^ijk^
11	Governor	2.95 ± 1.49^defghi^	4.30 ± 2.8^abcd^	0.27 ± 0.15^hijklm^	0.10 ± 0.11^bcd^	0.24 ± 0.31^defghijk^
12	Hua Su	2.00 ± 0.24^fghi^	3.8 ± 0.25^abcdef^	0.13 ± 0.03^klm^	0.07 ± 0.02^bcd^	0.45 ± 0.04^cdefgh^
13	Puff pastry	4.16 ± 0.62^cdefghi^	2.62 ± 0.44^abcdefghi^	0.45 ± 0.07^defghijklm^	0.18 ± 0.06^abcd^	0.36 ± 0.11^cdefghijk^
14	Foal selection	4.8 ± 0.84^cdefghi^	5.11 ± 0.23^ab^	0.17 ± 0.01^ijklm^	0.19 ± 0.08^abcd^	0.75 ± 0.06^bc^
15	Torch pear	8.56 ± 4.59^bcdef^	2.76 ± 1.12^abcdefghi^	0.51 ± 0.23^cdefghijklm^	0.31 ± 0.19^a^	0.43 ± 0.24^cdefghi^
16	Weining sour green pear	6.42 ± 2.58^bcdefghi^	2.87 ± 1.36^abcdefghi^	0.84 ± 0.35^cdefghi^	0.01 ± 0.01^d^	0.26 ± 0.13^defghijk^
17	Redskin	6.15 ± 0.07^bcdefghi^	3.12 ± 0.41^abcdefghi^	0.34 ± 0.02^fghijklm^	0.05 ± 0.01^bcd^	0.37 ± 0.16^cdefghijk^
18	Red Sun	2.38 ± 0.03^efghi^	0.81 ± 0.12^ghi^	0.38 ± 0.08^efghijklm^	0.008 ± 0.006^d^	0.02 ± 0.006^jk^
19	Evening show	3.43 ± 0.23^defghi^	3.59 ± 0.02^abcdefgh^	0.30 ± 0.007^ghijklm^	0.10 ± 0.01^abcd^	0.17 ± 0.01^ghijk^
20	Conde pear	1.74 ± 0.73^fghi^	0.94 ± 0.45^fghi^	0.12 ± 0.06^klm^	0.01 ± 0.01^abcd^	0.05 ± 0.02^hijk^
21	O'Woo pear	1.53 ± 0.61^ghi^	1.63 ± 0.59^defghi^	0.12 ± 0.05^klm^	0.01 ± 0.004^d^	0.12 ± 0.03^ghijk^
22	Gyokuro incense	4.39 ± 0.54^cdefghi^	1.54 ± 0.16^defghi^	0.26 ± 0.0^hijklm^	0.01 ± 0.004^d^	0.08 ± 0.05^hijk^
23	Gion	6.35 ± 2.13^bcdefghi^	2.74 ± 2.20^abcdefghi^	0.79 ± 0.44^cdefghijk^	0.08 ± 0.25^bcd^	0.21 ± 0.01^efghijk^
24	Rather early honey	1.13 ± 0.34^hi^	1.55 ± 0.14^defghi^	0.06 ± 0.02^m^	0.03 ± 0.01^bcd^	0.12 ± 0.04^ghijk^
25	Late Sanji	3.81 ± 0.40^defghi^	3.97 ± 0.11^abcde^	0.28 ± 0.02^hijklm^	0.22 ± 0.01^abc^	0.49 ± 0.12^cdefg^
26	Jin Shuiqiu	2.97 ± 0.41^defghi^	1.71 ± 0.05^defgh^i	0.20 ± 0.01^hijklm^	0.03 ± 0.05^bcd^	0.24 ± 0.04^defghijk^
27	Showa	4.93 ± 3.29^cdefghi^	2.56 ± 1.56^abcdefghi^	0.99 ± 0.65^bcdefg^	0.02 ± 0.02^cd^	0.08 ± 0.05^ghijk^
28	Longevity	3.75 ± 3.01^defghi^	2.88 ± 2.02^abcdefghi^	0.14 ± 0.10^jkl^m	0.10 ± 0.05^abcd^	0.42 ± 0.25^cdefghij^
29	Cangwu large sand pear	8.44 ± 1.62^bcdefg^	1.75 ± 0.2^cdefghi^	0.37 ± 0.08^efghijklm^	0.05 ± 0.04^bcd^	0.10 ± 0.04^ghijk^
30	Morning crisp	4.19 ± 2.04^cdefghi^	3.04 ± 1.1^abcdefghi^	0.30 ± 0.14^ghijklm^	0.02 ± 0.01^d^	0.61 ± 0.27^bcde^
31	Big duck pear	5.46 ± 2.41^cdefghi^	1.96 ± 0.6^cdefghi^	0.55 ± 0.21^cdefghijklm^	0.009 ± 0.004^d^	0.16 ± 0.07^ghijk^
32	Rendezvous big duck pear	3.98 ± 0.12^defghi^	1.51 ± 0.2^defghi^	0.45 ± 0.04^defghijklm^	0.02 ± 0.002^cd^	0.06 ± 0.008^hijk^
33	Large white pear	2.87 ± 1.98^defghi^	1.23 ± 0.8^efghi^	0.29 ± 0.18^ghijklm^	0.04 ± 0.03^bcd^	0.38 ± 0.26^cdefghijk^
34	Atago	2.78 ± 2.43^defghi^	2.65 ± 0.4^abcdefghi^	0.16 ± 0.07^ijklm^	0.02 ± 0.004^cd^	0.10 ± 0.02^ghijk^
35	Snow new	3.75 ± 2.11^defghi^	2.40 ± 0.6^abcdefghi^	0.32 ± 0.13^fghijklm^	0.02 ± 0.02^cd^	0.05 ± 0.03^hijk^
36	Moon	1.62 ± 0.86^fghi^	0.95 ± 0.8^fghi^	0.12 ± 0.06^klm^	0.08 ± 0.04^bcd^	0.21 ± 0.11^efghijk^
37	The old widow of Bin County	5.20 ± 0.46^cdefghi^	2.66 ± 0.1^abcdefghi^	0.78 ± 0.09^cdefghijk^	0.03 ± 0.01^bcd^	0.30 ± 0.05^defghijk^
38	Willow River Yellow Perfume	7.27 ± 2.17^bcdefghi^	2.32 ± 0.6^abcdefghi^	0.51 ± 0.11^cdefghijklm^	0.06 ± 0.02^bcd^	0.30 ± 0.11^defghijk^
39	Early crown	3.32 ± 0.11^defghi^	2.51 ± 0.3^abcdefghi^	0.27 ± 0.009^hijklm^	0.05 ± 0.02^bcd^	0.12 ± 0.03^ghijk^
40	Large pear water	6.87 ± 1.32^bcedfghi^	1.45 ± 0.1^defghi^	0.73 ± 0.08^cdefghijklm^	0.05 ± 0.03^bcd^	0.05 ± 0.01^hijk^
41	Joaquin	2.04 ± 0.26^fghi^	2.02 ± 0.02^cdefghi^	0.17 ± 0.11^ijklm^	0.02 ± 0.01^cd^	0.03 ± 0.03^ijk^
42	Xiangyuan pear	11.00 ± 0.72^abc^	2.83 ± 0.15^abcdefghi^	1.02 ± 0.17^bcdef^	0.01 ± 0.004^d^	0.29 ± 0.12^defghijk^
43	Morning crispy	4.06 ± 1.5^cdefghi^	2.23 ± 0.9^bcdefghi^	0.31 ± 0.1^ghijklm^	0.04 ± 0.04^bcd^	0.12 ± 0.05^ghijk^
44	Yaqing	1.02 ± 1.02^i^	0.85 ± 0.0.64^fghi^	0.08 ± 0.09^ghijklm^	0.02 ± 0.009^cd^	0.02 ± 0.01^jk^
45	Red sweet pears	1.69 ± 0.70^fghi^	1.61 ± 0.16^defghi^	0.27 ± 0.02^hijklm^	0.031 ± 0.01^cd^	0.03 ± 0.01^jk^
46	Green Skin	8.46 ± 3.28^bcdefg^	2.92 ± 0.52^abcdefghi^	1.11 ± 0.10^bcd^	0.01 ± 0.009^d^	0.12 ± 0.18^ghij^
47	Jinchuan wild pear	0.69 ± 0.08^i^	1.42 ± 0.31^defghi^	0.14 ± 0.02^ijklm^	0.11 ± 0.04^abcd^	0.05 ± 0.02^hijk^
48	Red pear	9.49 ± 2.99^bcd^	4.69 ± 1.81^abc^	1.05 ± 0.24^bcde^	0.13 ± 0.12^abcd^	0.02 ± 0.03^k^
49	Pear	5.24 ± 0.33^cdefghi^	2.20 ± 0.18^bcdefghi^	0.57 ± 0.03^cdefghijklm^	0.05 ± 0.07^bcd^	0.13 ± 0.06^ghijk^
50	Jin County big Ya pear	0.87 ± 0.09^i^	2.20 ± 0.18^bcdefghi^	0.07 ± 0.01^lm^	0.05 ± 0.03^bcd^	0.24 ± 0.01^defghijk^
51	De Sheng Xiang	3.15 ± 2.75^defgh^	1.60 ± 1.42^defghi^	0.16 ± 0.14^ijklm^	0.05 ± 0.05^bcd^	0.14 ± 0.12^ghijk^
52	Sister pear	4.99 ± 1.57^cdefghi^	3.72 ± 1.15^abcdefg^	0.31 ± 0.08^ghijklm^	0.12 ± 0.01^abcd^	0.97 ± 0.14^b^
53	Fragrant hemp	4.36 ± 1.09^cdefghi^	2.14 ± 0.09^cdefghi^	1.11 ± 0.07^bc^	0.08 ± 0.01^bcd^	0.11 ± 0.03^ghijk^
54	August Red	8.02 ± 0.74^bcdefgh^	3.30 ± 0.57^abcdefghi^	0.88 ± 0.05^bcdefgh^	0.11 ± 0.08^abcd^	1.42 ± 0.17^a^
55	Qingsong	3.46 ± 0.58^defghi^	1.52 ± 0.21^defghi^	0.29 ± 0.03^hijklm^	0.04 ± 0.004^bcd^	0.02 ± 0.02^jk^
56	Golden Autumn	1.52 ± 0.62^ghi^	3.34 ± 0.49^abcdefghi^	0.07 ± 0.02^lm^	0.02 ± 0.01^cd^	0.12 ± 0.01^ghijk^
57	Yellow incense	0.79 ± 0.17^i^	1.75 ± 0.41^cdefghi^	0.15 ± 0.03^ijklm^	0.06 ± 0.01^bcd^	0.04 ± 0.01^ijk^
58	nova	0.75 ± 0.14^i^	2.57 ± 0.12^abcdefghi^	0.08 ± 0.006^lm^	0.09 ± 0.008^bcd^	0.06 ± 0.01^hijk^
59	Hang Qing	3.81 ± 3.05^defghi^	1.65 ± 1.36^defgh^i	0.40 ± 0.32^efghijklm^	0.01 ± 0.01^d^	0.05 ± 0.03^hijk^
60	Long handful of ennifies	2.78 ± 1.03^defghi^	1.88 ± 0.34^cdefghi^	0.53 ± 0.14^cdefghijklm^	0.06 ± 0.01^bcd^	0.06 ± 0.006^hijk^
61	Small white pear	3.54 ± 0.54^defghi^	2.55 ± 0.26^abcdefghi^	0.77 ± 0.07^cdefghijkl^	0.01 ± 0.004^d^	0.07 ± 0.008^hijk^
62	Two slots	8.05 ± 7.35^bcdefgh^	2.26 ± 2.01^bcdefghi^	0.51 ± 0.46^cdefghijklm^	0.03 ± 0.03^cd^	0.14 ± 0.12^ghijk^
63	Linxia crisp pear	6.14 ± 3.06^bcdefghi^	1.63 ± 0.86^defghi^	0.70 ± 0.34^cdefghijklm^	0.06 ± 0.04^bcd^	0.10 ± 0.06^ghijk^
64	Zhongcui	0.73 ± 0.08^i^	1.17 ± 0.05^efghi^	0.10 ± 0.004^klm^	0.03 ± 0.006^cd^	0.08 ± 0.03^ghijk^
65	Apple pear	4.34 ± 2.69^cdefghi^	0.53 ± 0.28^i^	0.24 ± 0.13^hijklm^	0.02 ± 0.01^cd^	0.08 ± 0.05^hijk^
66	Old man pear	3.41 ± 0.46^defghi^	2.52 ± 0.58^abcdefghi^	0.22 ± 0.05^hijklm^	0.05 ± 0.01^bcd^	0.21 ± 0.04^efghijk^
67	Frozen fragrant pears	8.46 ± 1.93^bcdefg^	2.17 ± 0.66^bcdefghi^	0.37 ± 0.08^efghijklm^	0.16 ± 0.10^abcd^	0.35 ± 0.13^cdefghijk^
68	Shaydong black sour pear	2.81 ± 0.67^defghi^	0.64 ± 0.25^hi^	0.44 ± 0.07^defghijklm^	0.04 ± 0.006^bcd^	0.13 ± 0.05^ghijk^
69	Overwintering pears	1.43 ± 0.16^hi^	2.42 ± 0.11^abcdefghi^	0.30 ± 0.06^ghijklm^	0.12 ± 0.05^abcd^	0.10 ± 0.05^ghijk^
70	Green Cloud	3.50 ± 2.87^defghi^	2.13 ± 2.04^cdefghi^	0.19 ± 0.16^hijklm^	0.24 ± 0.35^ab^	0.22 ± 0.28^defghijk^
71	Lixian new Bapan	6.43 ± 1.59^bcdefghi^	3.81 ± 0.81^abcdef^	0.53 ± 0.14^cdefghijklm^	0.19 ± 0.04^abcd^	0.63 ± 0.11^bcd^
72	Mulberry pear	17.86 ± 4.50^a^	2.57 ± 0.30^abcdefghi^	1.57 ± 0.27^ab^	0.04 ± 0.03^bcd^	0.30 ± 0.04^defghijk^
73	Weining fragrant pear	1.14 ± 0.74^hi^	0.65 ± 0.31^hi^	0.14 ± 0.07^jklm^	0.02 ± 0.01^cd^	0.01 ± 0.01^k^
74	Great perfume	6.22 ± 0.24^bcdefghi^	2.99 ± 0.11^abcdefghi^	0.27 ± 0.09^hijklm^	0.07 ± 0.01^bcd^	0.37 ± 0.11^cdefghijk^
75	Golden pear	1.87 ± 1.62^fghi^	1.24 ± 0.84^efghi^	0.11 ± 0.09^klm^	0.04 ± 0.02^bcd^	0.10 ± 0.08^ghijk^
76	Green pear	4.60 ± 2.37^cdefghi^	2.16 ± 0.85^bcdefg^hi	0.58 ± 0.11^cdefghijklm^	0.06 ± 0.01^bcd^	0.10 ± 0.13^ghijk^
77	Gold pear	2.02 ± 0.74^fghi^	2.40 ± 0.15^abcdefghi^	0.09 ± 0.02^klm^	0.08 ± 0.05^bcd^	0.08 ± 0.03^ghijk^
78	Autumn perfume	9.13 ± 2.47^bcde^	2.00 ± 0.46^cdefghi^	0.36 ± 0.08^efghijklm^	0.07 ± 0.02^bcd^	0.14 ± 0.03^ghijk^
79	Botou big duck pear	4.67 ± 1.76^cdefghi^	2.41 ± 0.82^abcdefghi^	0.57 ± 0.20^cdefghijklm^	0.14 ± 0.07^abcd^	0.07 ± 0.03^hijk^

### 3.5 Antioxidant activity of different young pear fruits

The antioxidant activity of seventy-nine different young pear fruit varieties was assessed using the Ferric Reducing Antioxidant Power (FRAP) assay, revealing significant differences among the varieties (*P* < 0.05). The results, summarized in Table 2, indicated a broad range of total antioxidant capacities, measured in μmol Trolox/g FW, highlighting the diverse phytochemical profiles of these pear varieties. The FRAP values ranged from 1.315 ± 0.301 μmol Trolox/g FW to 0.126 ± 0.03 μmol Trolox/g FW. Varieties such as Lijian sesame pear, Red pear, Hua su, and Giao exhibited the highest FRAP activity, suggesting that these varieties possess superior antioxidant properties, potentially offering more significant health benefits due to their higher capacity to neutralize free radicals. Conversely, the lowest FRAP activity varieties included Weining fragrant pear, Jin county big ya pear, De sheng xiang, and Lixian new bapan, indicating a lower antioxidant capacity. (Sotiropoulos et al., 2016) also reported that the total antioxidant activity of two Greece-grown pear varieties (Naoussa and Vergina) measured by the FRAP method ranged from 1.41 mg AAE/g to 1.93 mg AAE/g, values slightly higher than those found in the present study. This discrepancy might be attributed to differences in pear varieties, extraction solvents, growing regions, and harvesting conditions. Additionally, (Wang et al., 2021) measured the antioxidant activity of the Josephine de Malines pear variety (*Pyrus communis*) using the FRAP assay and found a value of 4.37 mg AAE/g, which aligns more closely with values reported for Australian-grown pear varieties. The significant variation in antioxidant activity among the pear varieties underscores the influence of genetic and environmental factors on the phytochemical content of fruits. Understanding these differences is crucial for selecting enhanced nutritional and health benefits varieties. These findings can guide breeders and growers in developing and cultivating pear varieties with optimal antioxidant properties. In conclusion, the study demonstrates considerable variability in the antioxidant activity of seventy-nine young pear varieties, as measured by the FRAP assay. These differences reflect the impact of genetic diversity, growing conditions, and other environmental factors. The insights gained from this study are valuable for breeding programs and agricultural practices to improve the nutritional quality of pear fruits.

### 3.6 Multivariate analysis

**Fig. 2:**
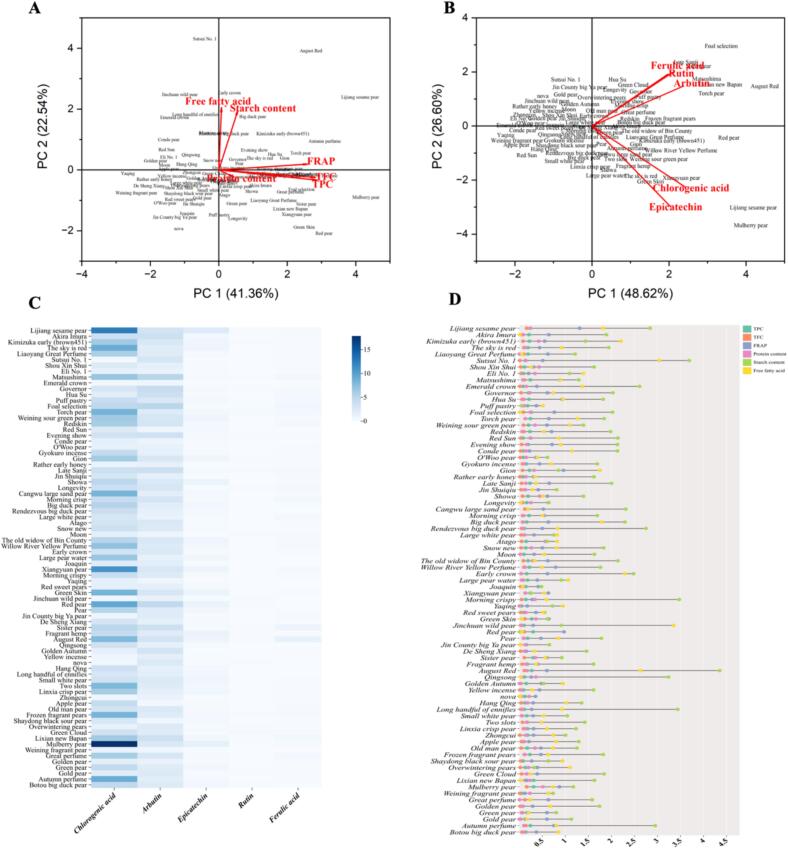
Multivariate analysis of starch, protein, free fatty acids, TPC, TFC, FRAP and nutritional value of seventy-nine different pear fruits varieties. A) Principal component analysis (PCA) of starch, protein, free fatty acids, TPC, TFC, and FRAP. B) Principal component analysis (PCA) based on the nutritional value of seventy-nine different pear fruits varieties. C) Heatmap of nutritional value. D) Dumbbell plot based on the starch, protein, free fatty acids, TPC, TFC, and FRAP of seventy-nine different pear fruits varieties.

The quantification of starch, protein, free fatty acids, TPC, TFC, FRAP, and other nutritional values among seventy-nine different pear fruits varieties were differentiated by the principal component analysis (PCA). PCA is widely used as dimentionality reduction technique that converts a set of original variables into a reduced set of uncorrelated variables known as principal components (PCs). Factor loading value, which represent the correlation of each variable (attribute) with the PCs, are used to identify the most significant variables and attributes within each dimention (Abdi & Williams, 2010). In the graphic, factor loading values are represented as vectors within a two-dimentional coordination system. Vectors of greater length suggest a strong correlation of the variable with the PC depicted along that axis. In this analysis, two principal components (PC1 and PC2) accounted for roughly 63.9 % of the total data variability, with PC1 explaining 41.36 % and PC2 22.54 % for the starch, protein, free fatty acids, TPC, TFC, and FRAP activity (Fig. 2A). The data presented in Fig. 2A showed that the starch and free fatty acids content was higher in Big duck pear, Early crown, August red, and Lijing sesame pear fruits varieties, while TPC, TFC and FRAP activity was higher in Touch pear, Autumn perfume, Liaoyang Great Perfume, and Mulberry pear fruits varieties. Similarly, PCA was used to summarized the similarities or differences among the nutritional values of seventy-nine different pear varieties, including Chlorogenic acid, Arbutin, Epicatechin, Rutin, and Ferulic acid (Fig. 2B). The combined contribution of the first two principal components (PC1 and PC2) explained 75.22 % of the total variability. Individually, PC1 accounted for 48.62 % of the variation, while PC2 explained 26.6 % of the datasets variability. Fig. 2B showed that the ferulic, rutin and arbutin content was higher in Late Sanji, Sister pear, Matsushima, Lixian new Bapan, August red, and Torch pear fruits varieties, while epicatechin, and chlorogenic acid content was higher in Lijing sesame pear, Mulberry pear, Xiangyuan pear, Green skin and Red skin pear fruits varieties. Overall, genotypes positioned to the right side of the along attribute vectors display higher nutritional value and antioxidant activity compared to those on the left. Interestingly, Fig. 2A and B showed that the antioxidant activity of Mulberry pear, Xiangyuan pear, Green skin pear fruits varieties might be due to the presence of high content of chlorogenic acid. This result is consistent with the findings of the Tang et al. (2019), who reported that the stronge antioxidant activity of tea extract attribute to the presence of chlorogenic acid. Further, heatmap analysis showed the nutraceutical composition of seventy-nine different pear fruits varieties (Fig. 2C). The data presented in Fig. 2C showed that the higher content of chlorogenic acid and arbitin was found in different pear fruits varieties while low content ferulic, rutin and epicatechin content was found in all seventy-nine pear fruits varieties. Similarly, dumbbell plot analysis was used to discriminate the seventy-nine different pear fruits varieties based on the composition of starch, protein, free fatty acids, TPC, TFC, and FRAP activity (Fig. 2D). The data given in Fig. 2D, showed that the higher starch and free fatty acids content was found in all pear fruits varieties, while low TFC value was found in all pear fruits varieties. These findings are consistence with the results reported by Du Toit et al. (2018). Interestingly, the antioxidant activity of Lijing sesame pear, Mulberry pear, Xiangyuan pear, Gion, Green skin and Red skin pear fruits varieties might be due to the high content of TPC. These findings are consistant with the findings of Mueed et al. (2023), who reported that high antioxidant and antimicrobial activities of medicinal plant extracts are attributed to the high content of TPC in plant extracts.

### 3.7 Molecular docking

**Fig. 3:**
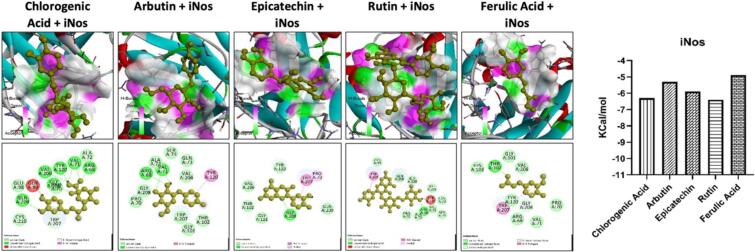
The molecular docking analysis of the ligand with protein target, i.e., iNOS. The ligand with the best docking energies such as Chlorogenic Acid, Arbutin, Epi-catechin, Rutin, and Ferulic Acid. The ligands showed variable interaction with the iNOS via multiple hydrogens and hydrophobic bonds. The binding energies of all the ligands are shown.

**Fig. 4:**
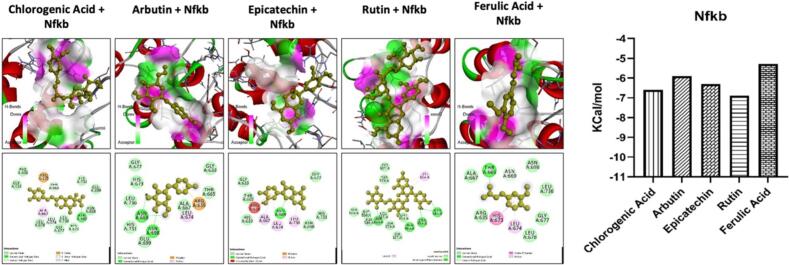
The molecular docking analysis of the ligand with protein target, i.e., NF-κB. The ligand with the best docking energies such as Chlorogenic Acid, Arbutin, Epi-catechin, Rutin, and Ferulic Acid. The ligands showed variable interaction with the NF-κB via multiple hydrogens and hydrophobic bonds. The binding energies of all the ligands are shown.

**Fig. 5:**
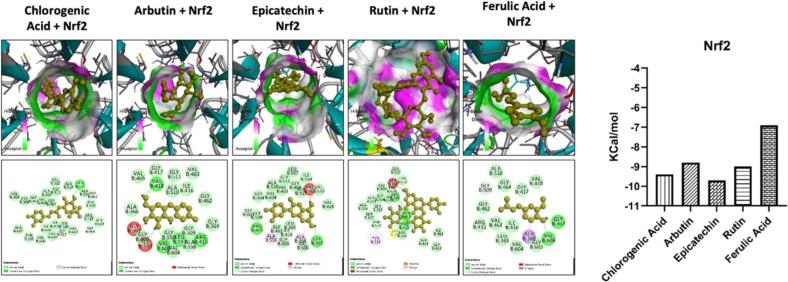
The molecular docking analysis of the ligand with protein target, i.e., Nrf2. The ligand with the best docking energies such as Chlorogenic Acid, Arbutin, Epi-catechin, Rutin, and Ferulic Acid. The ligands showed variable interaction with the Nrf2 via multiple hydrogens and hydrophobic bonds. The binding energies of all the ligands are shown.

Our study involved conducting molecular docking analysis on the constituents of *Pyrus communis,* focusing on Nrf2, NF-κB, and iNOS proteins. Through the molecular docking studies, we observed that chlorogenic acid, epicatechin, rutin, and ferulic acid exhibited a strong affinity towards these proteins (Fig. 3, Fig. 4, Fig. 5). Furthermore, these compounds formed hydrogen and hydrophobic bonds with Nrf2, NF-κB, and iNOS. We utilized computational tools to assess the Pharmacokinetic and Toxicokinetic profiles of the constituents found in the extract of *Pyrus communis*. While most compounds showed no toxicity in the studied models, they did display a diverse range of pharmacokinetic properties. The molecular docking analysis showed a significant interaction between the chlorogenic acid, arbutin, epicatechin, rutin, and ferulic acid with the Nrf2, iNOS, and NF-κB proteins. Additionally, the in-silico analysis portrayed diverse Pharmacokinetic and Toxicokinetic properties of the *Pyrus communis* constituents. In recent years, researchers have increasingly turned to computational analysis for swiftly screening numerous compounds and evaluating their biological properties. This approach provides valuable insights into a drug's interaction with its protein target and Pharmacokinetic and Toxicokinetic profile (Ahmed & Alkali, 2019).

## 4 Conclusion

The current study highlights the substantial metabolic and nutritional diversity among seventy-nine young pear fruit varieties, emphasizing the significant variability in total phenolic, flavonoid, and antioxidant activity. The observed differences are attributed to genetic diversity and environmental influences, underscoring the complexity of phytochemical compositions within pear species. Multivariate variate analysis showed that mulberry pear, Xiangyuan pear, Green skin pear fruits cultivars detected with highest neutritional value and antioxidant activity, respectively. Molecular docking studies further suggested that all bioactive compounds (chlorogenic acid, epicatechin, rutin, and ferulic acid) in pears fruit exhibit strong interactions with essential proteins, indicating potential health benefits. These insights are crucial for breeders aiming to develop varieties with enhanced nutritional properties and for the food industry to optimize pear cultivation and processing practices. Future research should explore bioactive compound accumulation's genetic and environmental determinants in pears to improve their dietary and health benefits.

## Funding

This research was financially supported by the 10.13039/100007540Jiangsu Agricultural Science and Technology Innovation Fund [CX(22)2025], the Agriculture Research System of China (CARS-28).

## CRediT authorship contribution statement

**Abdul Basit:** Writing – review & editing, Writing – original draft, Methodology, Data curation, Conceptualization. **Abdul Mueed:** Writing – review & editing. **Li Min:** Writing – review & editing. **Niu Mingxu:** Writing – review & editing. **Gong Xin:** Writing – review & editing. **Raheem Shahzad:** Writing – review & editing. **Wen Yue:** Writing – review & editing. **Tian Jia:** Writing – review & editing. **Tao Shutian:** Writing – review & editing, Writing – original draft, Visualization, Supervision, Project administration, Methodology, Investigation, Funding acquisition, Data curation, Conceptualization.

## Declaration of competing interest

“The authors declare that they have no known competing financial interests or personal relationships that could have appeared to influence the work reported in this paper.”

## Data Availability

No data was used for the research described in the article. The original data contributions are present within the article, and the data can be disseminated upon the formal request.
